# Insights from a web-based questionnaire: examining diagnostic procedures prior to magnetic resonance imaging

**DOI:** 10.1186/s13584-024-00636-6

**Published:** 2024-09-18

**Authors:** Jacob Sosna

**Affiliations:** Department of Radiology, Faculty of Medicine, Hadassah Medical Center, Hebrew University of Jerusalem, Jerusalem, 91120 Israel

## Abstract

The appropriate use of diagnostic imaging, particularly MRI, is a critical concern in modern healthcare. This paper examines the current state of MRI utilization in Israel, drawing on a recent study by Kaim et al. that surveyed 557 Israeli adults who underwent MRI in the public health system. The study revealed that 60% of participants had undergone other imaging tests before their MRI, with 23% having more than one prior examination. While these findings highlight potential inefficiencies in the diagnostic pathway, they also underscore the complexity of medical decision-making in imaging.

The paper discusses various factors influencing MRI utilization, including regulatory pressures, healthcare system structure, and the need for evidence-based guidelines. It explores potential strategies for optimizing MRI justification and scheduling, such as implementing clinical decision support systems, enhancing interdisciplinary communication, and leveraging artificial intelligence (AI) for predictive analytics and resource optimization.

The need for comprehensive research into MRI justification and scheduling optimization is presented. Key areas for investigation include the effectiveness of decision support tools, patient outcomes, economic analyses, and the application of quality improvement methodologies.

Ensuring the appropriate use of diagnostic imaging, is a critical concern in modern healthcare. There is growing recognition of the need to justify each exam based on clinical necessity. Retrospective studies have found inappropriate CT and MRI use rates ranging from 10 to 39% when referrals are assessed against clinical guidelines [1–5]. As overall imaging utilization continues to rise rapidly due to technological advancements, increasing population and expanding indications, optimizing the appropriateness of each exam becomes increasingly vital.

The justification and optimization of medical imaging is crucial for patient safety while delivering clinical benefits. Ensuring each exam is truly medically indicated based on presenting symptoms or diagnostic questions is paramount at the population level.

In this issue Kaim et al. [6] conducted an online survey of patient experience of scheduling an MRI among 557 Israeli adults, that underwent an MRI in the public health system within the past year. They have shown that that 60% of participants underwent other imaging tests before their MRI scan. Of those, computed tomography (CT) scans (43%), X-rays (39%), and ultrasounds (32%) were the most common additional imaging procedures. In addition, of the 60% of participants, 23% had undergone more than one prior imaging examination.

The cascade in which imaging studies are performed is complex. A patient may present with signs and symptoms that highlight a need for further imaging. It may start with indicationX-Rays or ultrasound such as in the case of orthopedic trauma and based on the findings such as suspected meniscal injury further require advanced studies such as an MRI. Another possibility is directly performing an MRI study when there is clear indication such as acute neurological symptoms with suspected spinal injury.

The results by Kaim et al. represent the current situation in Israel but does not enable at this stage analysis of the reasons for this phenomenon. On one hand it may be possible that too many patients underwent prior non-MRI imaging studies because of difficulties in getting an approval or scheduling an appointment for an MRI study. In this scenario direct referral for an MRI may have been more beneficial. Another possibility is that prior studies results indicated the need for further evaluation with MRI and from a medical perspective this was the correct process to arrive at a diagnosis. A patient with undetermined liver lesion on CT or US may necessitate an MRI for diagnosis but this should only be performed as a problem-solving study. Follow-up of prior medical conditions such as multiple sclerosis necessitates serial MRI studies and the lack of separation of initial diagnostic and follow-up studies further complicates the analysis on the appropriateness of MRI studies. The study by Kaim et al. did not analyze the details of the referrals and the diagnoses of the MRI studies and therefore did not do a root cause analysis of the possible reasons.

## Health system implications

The health system in Israel is under pressure in recent decades to increase its efficiency and to use its resources in an optimized way in order to promote health. Our system is heavily regulated and centralized with only four HMO’s with each one with its own approval pathway for advanced imaging studies. Imaging equipment is also regulated with the need for a Certificate of Need approval for each MRI and CT scanner.

The study by Kaim et al. raises insightful observations about a potentially significant issue in healthcare resource allocation and diagnostic practices. If there’s a shortage of MRI scanners, healthcare providers might indeed resort to CT scans as an interim measure. This could lead to increased radiation exposure for patients, as CT scans use ionizing radiation while MRI does not. It may also lead to potential misdiagnosis or delayed diagnosis, as MRI is superior for certain conditions, especially soft tissue injuries. Cost implications are also an issue as CT scans are generally less expensive than MRIs, but multiple CT scans while waiting for an MRI could end up being more costly overall. Workflow inefficiencies may also occur as performing CT scans as a stopgap measure could create duplicate work and strain radiology departments.

Patient experience might be affected as multiple imaging tests could lead to patient frustration, anxiety, and inconvenience.

Taking into account all these considerations might have long-term health policy implications which could drive investments in increased MRI scanners and training of specialists.

## Clinical justification

The means to improve justification of MRI studies include evidence-based guidelines such as the American College of Radiology or iGuide which is a Europeanized version of these guidelines. This can be achieved with implementing electronic systems that guide clinicians in selecting appropriate imaging studies. Education and training are extremely important in optimizing the process. These may include regular updates for referring physicians on appropriate use criteria and training on risks and benefits of different imaging modalities [7–9]. Alternatives assessment is always an issue. Should the referring physician consider less expensive (CT) or non-radiation alternatives (MRI) when appropriate. Assessment of previous imaging results could suffice is also an important solution. In some patients assessed by Kaim et al. this could have been the case for prior imaging studies.

Collaborative decision-making can make the medical system more efficient. Direct communication between referring physicians and radiologists may optimize the selection and timing of the needed imaging studies. Involving patients in the decision-making process can also be beneficial.

Regulatory bodies may use audit and feedback for regular reviews of imaging referral patterns and provide feedback to clinicians on their referral appropriateness. Cost-effectiveness considerations should evaluate the potential clinical impact versus the cost of the study as well as societal and healthcare system resource allocation.

## The role of AI

AI can play a significant role in optimizing the justification of MRI studies for patients. Some key applications may include AI-powered systems that can analyze patient data, symptoms, and medical history to suggest appropriate imaging studies. These systems can provide real-time guidance to clinicians, helping them make more informed decisions about ordering MRI scans. AI algorithms can predict the likelihood of an MRI study yielding clinically significant results based on patient characteristics and symptoms. This can help prioritize patients who are most likely to benefit from an MRI.

Using natural language processing (NLP) AI can analyze unstructured clinical notes and radiology reports to extract relevant information. This can help in assessing the appropriateness of previous imaging studies and avoiding unnecessary repeat scans. Outcome prediction is also of interest as AI models can predict the potential impact of an MRI study on patient management and outcomes. This can help in justifying the need for the study, especially in cases where the clinical benefit is not immediately apparent.

Resource optimization is also needed and can be achieved with AI. AI can analyze scheduling patterns and patient flow to optimize MRI utilization and reduce wait times. This can help in justifying urgent studies and managing resource allocation more effectively.

## Research implications

Justification and optimization of MRI scheduling for patients is a crucial area with significant potential for improving healthcare delivery, patient outcomes, and resource utilization. Some key areas where research could be valuable include effectiveness of decision support tools, patient outcomes, economic analyses, predictive modeling and patient-centered scheduling. Quality improvement methodologies can help to study the effectiveness of various quality improvement approaches (e.g., Lean, Six Sigma) in optimizing MRI scheduling processes. Comparative effectiveness research is needed for comparing different MRI scheduling and justification strategies across various healthcare systems and patient populations and help identifying best practices that can be widely adopted.

One time point assessment is not sufficient. The study by Kaim et al. raises important issues and should be expanded to fully understand the use of MRI in Israel. Long-term impact assessment can be performed with longitudinal studies to evaluate the long-term effects of optimized MRI scheduling and patterns of use on healthcare system efficiency and patient outcomes. This is highly recommended on a nationwide basis.

## Conclusions

Appropriate use of diagnostic imaging is crucial in modern healthcare. A survey in Israel revealed that 60% of patients underwent other imaging tests before their MRI, with CT scans, X-rays, and ultrasounds being the most common. The reasons for multiple imaging studies are complex and may include difficulties in scheduling MRI appointments, medical necessity, or follow-up requirements for specific conditions.

Further research is needed in areas such as the effectiveness of decision support tools, patient outcomes, economic analyses, and comparative effectiveness across healthcare systems.

## Data Availability

Not applicable.

## Abbreviations

AI: Artificial intelligence
NLP: Natural language processing
CT: Computerized tomography
MRI: Magnetic resonance imaging
